# Dual-Energy CT-Derived Parameters: A Promising Tool for Noninvasive Prediction of Glypican-3 in Hepatocellular Carcinoma

**DOI:** 10.3390/diagnostics16060850

**Published:** 2026-03-12

**Authors:** Yiwan Guo, Fan Pu, Jinrong Yang, Aiping Yang, Ying Yang, Ruiyao Tang, Xin Li, Fan Yang

**Affiliations:** 1 Department of Radiology, Union Hospital, Tongji Medical College, Huazhong University of Science and Technology, Jiefang Avenue # 1277, Wuhan 430022, China; 2 Hubei Provincial Clinical Research Center for Precision Radiology & Interventional Medicine, Wuhan 430022, China; 3 Hubei Key Laboratory of Molecular Imaging, Wuhan 430022, China

**Keywords:** hepatocellular carcinoma, Glypican-3, dual-energy CT, imaging biomarker, noninvasive prediction

## Abstract

**Background/Objectives:** Glypican-3 (GPC3), a membrane-bound heparan sulfate proteoglycan, has been identified as a promising target for both the diagnosis and treatment of hepatocellular carcinoma (HCC). However, the diagnosis of GPC3 expression mainly depended on invasive procedures. This study aimed to investigate the potential of dual-energy computed tomography (DECT)-derived parameters for noninvasive prediction of GPC3 expression in HCC. **Methods:** This retrospective study included 79 HCC patients with confirmed GPC3 immunohistochemistry and pretreatment contrast-enhanced DECT. Qualitative imaging features and quantitative DECT parameters, including iodine density of HCC (ID_Ca_), normalized iodine density (NID), slope of spectral attenuation curve (λ_HU_), and effective atomic number (Z_eff_), were evaluated in both arterial and portal venous phases. Univariate and multivariate logistic regression analyses were employed to identify independent predictors, and a combined model was subsequently constructed. Receiver operating characteristic (ROC) curve analysis was performed to assess the diagnostic efficiency of imaging parameters in predicting GPC3 expression. Interobserver agreement of DECT parameters was evaluated using intraclass correlation coefficients (ICC). **Results:** GPC3-positive HCCs demonstrated significantly higher arterial phase (AP) ID_Ca_, NID, λ_HU_, and Z_eff_ (all *p* ≤ 0.001) than GPC3-negative HCCs. Multivariate logistic regression analysis identified NID-AP [Odds ratio (OR) = 2.00, *p* = 0.010] and peritumoral enhancement (OR = 9.25, *p* = 0.046) as independent predictors. The model combining NID-AP and peritumoral enhancement achieved the best diagnostic performance (AUC = 0.781, sensitivity = 67.86%, specificity = 78.26%) for predicting GPC3 expression. All DECT-derived parameters showed excellent interobserver reproducibility (ICC > 0.75 for all). **Conclusions:** Parameters derived from DECT, especially combining NID-AP and peritumoral enhancement, may be a potential tool to noninvasively predict GPC3 expression in HCC.

## 1. Background

Hepatocellular carcinoma (HCC) is the most common primary liver malignancy and remains one of the leading causes of cancer-related mortality worldwide [1]. Glypican-3 (GPC3), a membrane-bound heparan sulfate proteoglycan, has emerged as a critical biomarker for HCC. It is overexpressed in malignant hepatic tissues but absent in normal and cirrhotic livers, rendering it a promising target for both diagnosis and therapy [2].

GPC3 plays a pivotal role in HCC progression by modulating several key signaling pathways, including Wnt/β-catenin, insulin-like growth factor, and Hedgehog pathways [3,4]. Its overexpression is correlated with aggressive tumor behavior, vascular invasion, and unfavorable prognosis [5]. Consequently, GPC3 has been investigated as a therapeutic target, with multiple immunotherapeutic strategies under development, including monoclonal antibodies, peptide vaccines, and chimeric antigen receptor (CAR) T-cell therapies [6,7,8].

In clinical practice, GPC3 expression is primarily evaluated through invasive procedures, such as immunohistochemical analysis of biopsy or surgical specimens [9]. These methods carry inherent risks, such as bleeding and sampling errors, and may not be suitable for all patients. Serum GPC3 measurements provide a less invasive alternative. However, they exhibit limited sensitivity and specificity [10]. Therefore, there is an urgent need for non-invasive, accurate, and reliable approaches to assess GPC3 expression in HCC patients.

Dual-energy computed tomography (DECT) has emerged as a promising imaging technique, providing quantitative parameters that enhance tissue characterization beyond conventional CT. By acquiring images at two distinct energy levels, DECT enables material differentiation based on differences in atomic number and X-ray attenuation, allowing for more precise tissue analysis [11]. DECT-derived parameters, including iodine density and effective atomic number, have been employed to evaluate tumor vascularity and heterogeneity [12,13], which may correlate with molecular markers such as GPC3. Despite its potential, the application of DECT for predicting GPC3 expression in HCC has not been extensively investigated.

In light of these considerations, this study aimed to evaluate the value of using DECT-derived quantitative parameters to noninvasively predict GPC3 expression in HCC.

## 2. Methods

### 2.1. Study Patients

**Figure 1:**
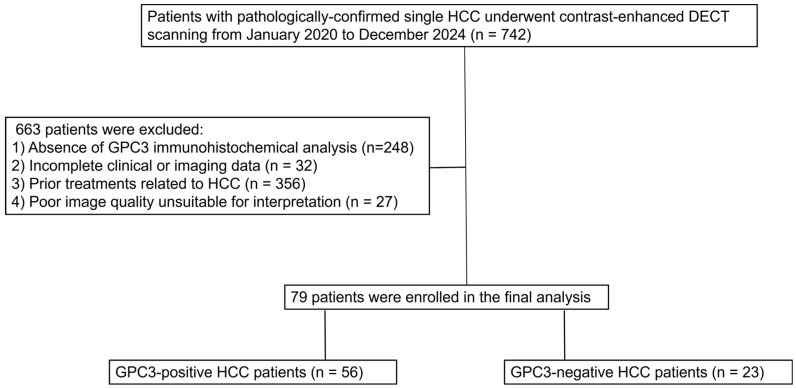
Flowchart of patient selection. HCC, hepatocellular carcinoma; DECT, dual-energy CT; GPC3, glypican-3.

Approval for this retrospective study was granted by the Ethics Committee of Union Hospital, Tongji Medical College, Huazhong University of Science and Technology (Approval Number: UHCT250163; approval date: 27 February 2025). The study was conducted in accordance with the Declaration of Helsinki, and informed consent was waived. The inclusion criteria in this study were as follows: (1) age ≥ 18 years; (2) a single HCC lesion histopathologically confirmed by surgery; and (3) contrast-enhanced DECT performed within four weeks prior to treatment. The exclusion criteria were as follows: (1) absence of GPC3 immunohistochemical analysis; (2) incomplete clinical or imaging data; (3) prior treatments related to HCC, including radiofrequency ablation, transarterial chemoembolization (TACE), systemic therapy, or hepatectomy; and (4) poor image quality unsuitable for interpretation. The study flowchart of patient selection is shown in Figure 1.

### 2.2. Clinicopathological Analyses

Baseline clinicopathological data, including demographics, etiology, cirrhosis, tumor size, Edmondson–Steiner grade, macrovascular invasion, and key laboratory indicators [alanine aminotransferase (ALT), aspartate aminotransferase (AST), total bilirubin (TBIL), albumin (ALB), serum creatinine (SCr), platelet count (PLT), prothrombin time (PT), international normalized ratio (INR), and alpha-fetoprotein (AFP)], were retrospectively obtained. A pathologist with over 10 years of experience in liver pathology conducted the histopathological and immunohistochemical analyses. To precisely evaluate GPC3 expression, we employed the scoring scale developed by Takai et al. [14], which accounted for both the proportion of positive tumor cells and their staining intensity. The positive cell rates were classified into four grades: 0 (<5% positive tumor cells), 1+ (5–10% positive tumor cells), 2+ (10–50% positive tumor cells), and 3+ (>50% positive tumor cells). Staining intensity was categorized as weak, moderate, or strong. Consistent with previous studies [15,16], GPC3 expression was considered negative if the positive cell rate was grade 0 (irrespective of staining intensity) or grade 1+ with weak staining.

### 2.3. CT Acquisition

CT scans were acquired using a dual-layer spectral CT scanner (IQon; Philips Healthcare, Andover, MA, USA). The technical parameters were set as follows: tube voltage at 120 kVp; detector collimation of 64 × 0.625 mm; reconstructed slice thickness and interval both at 1.5 mm; helical pitch of 0.8; matrix size of 512 × 512; and rotation time lasting 0.5 s. Tube current was modulated automatically using Dose Right Index (version 22; Philips Healthcare). The scanning range extended from the top of the diaphragm to the inferior liver margin. An iodine-based contrast medium (iodixanol, 320 mg I/mL; Hengrui Healthcare, Suzhou, China) was administered intravenously at 1.2 mL/kg, followed by a 40 mL saline flush. Contrast-enhanced scanning was triggered using Smart Prep technology in the abdominal aorta at the level of the celiac trunk (threshold: 100 HU). The arterial phase (AP) scan commenced 8 s after reaching the threshold, and the portal venous phase (PVP) was initiated 24 s after the end of AP acquisition. Conventional images were generated with the iDose4 algorithm, whereas spectral-based images utilized a dedicated spectral reconstruction algorithm.

### 2.4. Image Analysis

Images were sent to a dedicated postprocessing workstation (Syngo via, Siemens Healthcare) for further analysis. Two radiologists (YWG and FP, with 5 and 3 years of experience in abdominal imaging, respectively) independently assessed the CT images without access to clinical and histological data. A third radiologist (XL, with over 20 years of experience in abdominal imaging) resolved any discrepancies. Imaging characteristics were evaluated using the Liver Imaging Reporting and Data System (LI-RADS) 2018 criteria [17] within the picture archiving and communication system (PACS), including: (1) arterial phase enhancement; (2) enhancing capsule; (3) nonperipheral washout; (4) targetoid appearance; (5) mosaic architecture; (6) nodule-in-nodule pattern; (7) intratumoral hemorrhage; (8) tumor margin; (9) peritumoral enhancement; (10) necrosis or severe ischemia; and (11) internal artery. Definitions of these features are provided in Table S1 according to the LI-RADS 2018 criteria.

**Figure 2:**
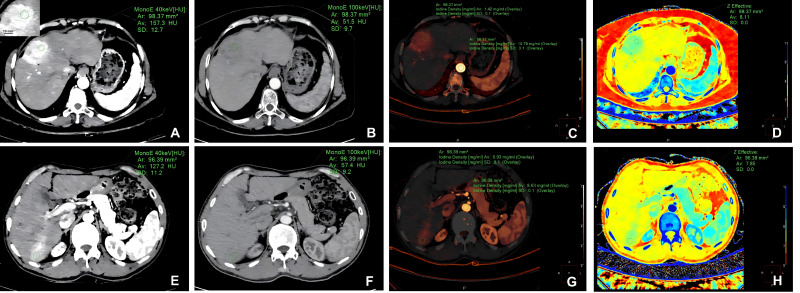
The typical cases of measuring DECT quantitative parameters in GPC3-positive and GPC3-negative groups. Regions of interest (maximum diameter ≤ 10 mm) were manually placed within hypervascular tumor areas, avoiding necrotic areas, large vessels, hemorrhage, and adjacent non-tumorous liver parenchyma (Scale bar = 10 mm) (**A**). 40 keV virtual monochromatic image in arterial phase (**A**), 100 keV virtual monochromatic image in arterial phase (**B**), iodine density overlay image in arterial phase (**C**), and effective atomic number image in arterial phase (**D**) of a GPC3-positive patient. 40 keV virtual monochromatic image in portal venous phase (**E**), 100 keV virtual monochromatic image in portal venous phase (**F**), iodine density overlay image in portal venous phase (**G**), and effective atomic number image in portal venous phase (**H**) of a GPC3-negative patient.

Reconstructed spectral CT images included virtual monochromatic images (VMIs) from 40 to 190 keV, effective atomic number (Z_eff_) images, and iodine-based material decomposition images. As the previous study did [18], regions of interest (ROIs) were manually placed within the solid and visually hypervascular portions of the tumor while systematically avoiding necrotic areas, large vessels, hemorrhage, and adjacent non-tumorous liver parenchyma. For each lesion, three ROIs (maximum diameter ≤ 10 mm) were placed on the maximum cross-section of the lesion and on its two adjacent sections. The mean value of these ROIs was used for statistical analysis. Additional ROIs were placed on the aorta at the same axial level. ROI placement was consistent across image types and phases. The following parameters were acquired during AP and PVP: CT attenuation values at 40 and 100 keV VMIs (HU_40keV_, HU_100keV_), iodine density of HCC (ID_Ca_) and aorta (ID_aorta_), and Z_eff_ of HCC. Normalized iodine density (NID) was calculated as NID = ID_Ca_/ID_aorta_. The slope of the spectral attenuation curve (λ_HU_) was calculated as (HU_40keV_–HU_100keV_)/60. The typical cases of measuring DECT quantitative parameters in GPC3-positive and GPC3-negative groups are shown in Figure 2. ROI placement and measurements were independently performed by two radiologists (YWG and FP), and interobserver reproducibility was evaluated using intraclass correlation coefficients (ICCs).

### 2.5. Statistical Analysis

All statistical analyses were performed using GraphPad Prism (version 10.1.2) and R software (version 4.4.3; URL https://www.r-project.org/, accessed on 15 August 2025). The Shapiro–Wilk test was used to assess the normality of data distribution. Normally distributed continuous variables were expressed as mean ± standard deviation, while non-normally distributed data were expressed as median with interquartile range. Student’s *t*-test or the Wilcoxon rank-sum test was used to compare continuous variables, and categorical variables were compared using the Chi-square test. To determine independent predictors of GPC3 expression, univariate and multivariate logistic regression analyses were conducted. Given the relatively small study sample size, variables with *p*-values < 0.1 in the univariate analysis were subsequently included in the multivariate analysis. Then, the combined model was constructed. Receiver operating characteristic (ROC) curve analysis was performed to assess the diagnostic efficiency of imaging parameters for predicting GPC3 expression. Decision curve analysis (DCA) was used to evaluate the clinical applicability of DECT parameters and the combined model by calculating net benefits across various threshold probabilities. Interobserver agreement of DECT parameters was evaluated using intraclass correlation coefficients (ICCs). A two-tailed *p*-value less than 0.05 indicated statistical significance.

## 3. Results

### 3.1. Baseline Clinicopathological Characteristics of Patients

A total of 79 patients with HCC were enrolled and categorized into GPC3-positive (*n* = 56) and GPC3-negative (*n* = 23) groups (Figure 1). There were no statistically significant differences between the two groups regarding age, gender, etiology, cirrhosis, tumor size, or Edmondson-Steiner grade (all *p* > 0.05). Several key laboratory parameters, including ALT, AST, TBIL, ALB, SCr, PLT, INR, PT, and AFP, also did not differ significantly between the two groups (Table 1).

### 3.2. Qualitative CT Imaging Features of HCC

Most qualitative CT features of HCC did not significantly differ between GPC3-positive and GPC3-negative patients, including arterial phase enhancement, internal artery presence, non-peripheral washout, enhancing capsule, targetoid appearance, mosaic architecture, nodule-in-nodule pattern, intratumoral hemorrhage, tumor margin, and necrosis or severe ischemia (all *p* > 0.05). Notably, peritumoral enhancement was significantly more prevalent in the GPC3-positive group (32.14%) compared to the GPC3-negative group (8.70%) (*p* = 0.029) (Table 2).

### 3.3. Comparison of Quantitative DECT Parameters Between the Two Groups

**Figure 3:**
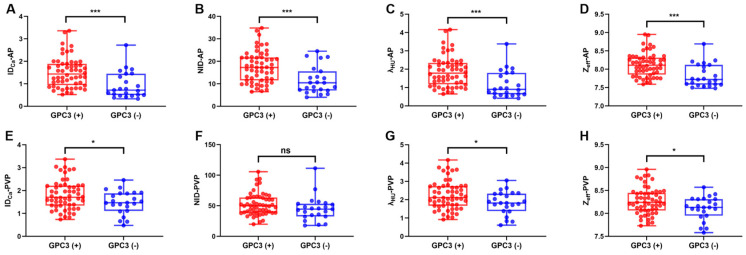
Comparison of quantitative DECT parameters between GPC3-positive and GPC3-negative groups. (**A**–**D**), comparison of arterial phase DECT parameters, including ID_Ca_-AP, NID-AP, λ_HU_-AP, and Z_eff_-AP, between these two groups; (**E**–**H**), comparison of portal venous phase DECT parameters, including ID_Ca_-PVP, NID-PVP, λ_HU_-PVP, and Z_eff_-PVP, between these two groups. AP, arterial phase; PVP, portal venous phase; ID_Ca_, iodine density of HCC; NID, normalized iodine density; λ_HU_, slope of spectral attenuation curve; Z_eff_, effective atomic number. ns not significant * *p* < 0.05 *** *p ≤* 0.001.

In the arterial phase, the GPC3-positive group demonstrated higher values of ID_Ca_, NID, λ_HU_, and Z_eff_ compared to the GPC3-negative group (all *p* ≤ 0.001). In the portal venous phase, significant differences were also observed in ID_Ca_, λ_HU_, and Z_eff_ (*p* = 0.028, 0.028, and 0.024, respectively) between the two groups, while the difference in NID was not statistically significant (*p* = 0.077) (Table 3; Figure 3).

### 3.4. Univariate and Multivariate Logistic Regression Analysis

As presented in Table 4, univariate logistic regression analysis revealed that arterial phase parameters including ID_Ca_ [Odds ratio (OR) = 5.34, *p* = 0.002], NID (OR = 1.16, *p* = 0.002), λ_HU_ (OR = 2.82, *p* = 0.005), and Z_eff_ (OR = 27.34, *p* = 0.001) were significantly associated with GPC3 positive expression. Portal venous phase parameters such as ID_Ca_, λ_HU_, and Z_eff_ were also statistically significant. Peritumoral enhancement was also associated with a higher odds ratio (OR = 4.97, *p* = 0.043). In the multivariate analysis, only NID-AP (OR = 2.00, *p* = 0.010) and peritumoral enhancement (OR = 9.25, *p* = 0.046) remained independent predictors of GPC3 expression.

### 3.5. Diagnostic Performance of DECT Parameters

**Figure 4:**
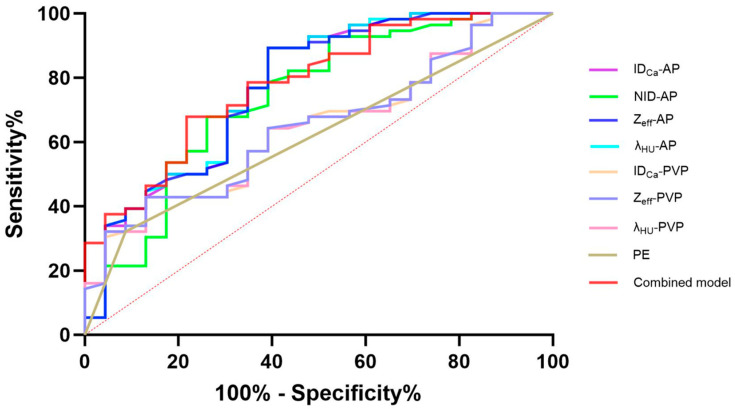
Receiver operating characteristic curves of quantitative and qualitative DECT parameters for predicting GPC3 expression of HCC patients. GPC3, glypican-3; AP, arterial phase; PVP, portal venous phase; ID_Ca_, iodine density of HCC; NID, normalized iodine density; λ_HU_, slope of spectral attenuation curve; Z_eff_, effective atomic number; PE, peritumoral enhancement; Combined model, the model combining NID-AP and peritumoral enhancement.

**Figure 5:**
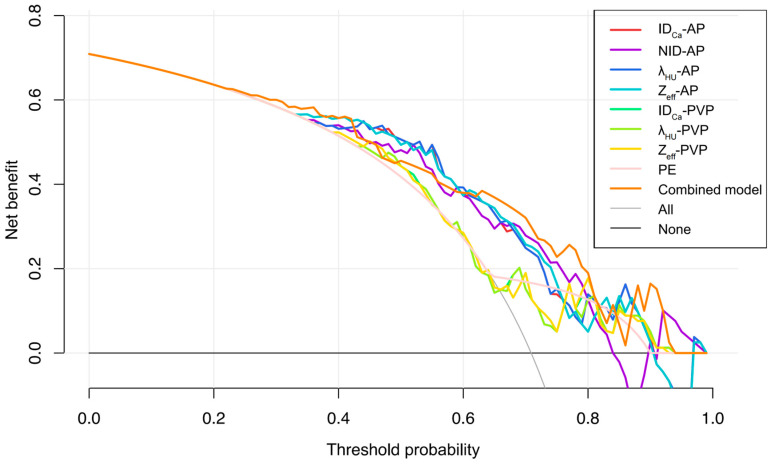
Decision curve analysis of DECT parameters and the combined model to predict GPC3-positive HCC. GPC3, glypican-3; AP, arterial phase; PVP, portal venous phase; ID_Ca_, iodine density of HCC; NID, normalized iodine density; λ_HU_, slope of spectral attenuation curve; Z_eff_, effective atomic number; PE, peritumoral enhancement; Combined model, the model combining NID-AP and peritumoral enhancement.

ROC curve analysis demonstrated good diagnostic performance of several arterial-phase DECT parameters for predicting GPC3 expression (Table 5; Figure 4). The AUC values for ID_Ca_-AP, NID-AP, λ_HU_-AP, and Z_eff_-AP were 0.774, 0.745, 0.775, and 0.772, respectively. In contrast, portal venous phase parameters had lower AUC values (0.645–0.647). The combined model incorporating NID-AP and peritumoral enhancement according to the results of multivariate logistic regression analysis achieved the highest diagnostic efficiency, with an AUC of 0.781, a sensitivity of 67.86%, and a specificity of 78.26%. However, the DeLong test suggested that no statistically significant differences were observed among the AUC values of the combined model and the quantitative DECT parameters in AP and PVP (all *p* > 0.05). Furthermore, decision curve analysis also confirmed satisfactory clinical benefits of the combined model (Figure 5).

### 3.6. Interobserver Agreement of Quantitative DECT Parameters

Interobserver reproducibility for DECT parameters was excellent. All intraclass correlation coefficients (ICCs) exceeded 0.75 (Table S2), indicating reliable measurements and high consistency between the two radiologists.

## 4. Discussion

In this study, we found that DECT-derived arterial phase parameters, specifically NID-AP and peritumoral enhancement, were independently associated with GPC3 expression in HCC. The combination of these features yielded favorable diagnostic performance (AUC = 0.781). Other DECT-derived metrics, including λ_HU_ and Z_eff_, also exhibited significant discriminative power, particularly during the arterial phase, highlighting the utility of DECT for noninvasive molecular characterization of HCC.

The observed differences in DECT parameters between GPC3-positive and GPC3-negative tumors may be related to underlying biological characteristics. GPC3 plays a central role in hepatocarcinogenesis by modulating oncogenic pathways such as Wnt/β-catenin, IGF, and Hedgehog, and has been shown to promote angiogenesis through upregulation of vascular endothelial growth factor (VEGF) [19]. This angiogenic activity may be a possible explanation for the elevated iodine density and steeper λ_HU_ slope observed in GPC3-positive tumors. The higher Z_eff_ values observed in GPC3-positive tumors may be associated with differences in tissue composition, consistent with their aggressive phenotype [20]. Furthermore, the significantly higher incidence of peritumoral enhancement in GPC3-positive lesions may reflect infiltrative growth patterns and reactive neovascularization at the tumor margin [21]. Nevertheless, the biological interpretations proposed above are intended to provide possible explanatory context for the observed imaging associations and should be interpreted as hypotheses rather than definitive mechanistic conclusions. Direct validation using histopathological or molecular correlates will be required in future studies.

Our findings are consistent with emerging evidence that quantitative imaging features may be associated with molecular characteristics in HCC. Specifically, arterial phase NID remained an independent predictor of GPC3 expression after multivariable analysis, supporting its potential as a surrogate biomarker. Notably, peritumoral enhancement, as an easily recognizable feature, was found to be associated with GPC3 expression in our study. Previous studies also suggested that peritumoral enhancement was more common in the GPC3-positive group [22], and it could be used to predict high-risk pathologic features and early recurrence of HCC [23].

Given the growing interest in GPC3 as a therapeutic target for antibody-based and immune checkpoint therapies, noninvasive prediction of its expression status has practical implications. While prior efforts have investigated GPC3-targeted molecular imaging using PET agents such as ^89^Zr-ssHN3 and [^68^Ga]Ga-RAYZ-8009, these techniques are limited by high cost, radiation exposure, and limited clinical accessibility [24,25]. Several MRI-based and radiomics models have been reported for noninvasive prediction of GPC3 expression, with AUCs in validation cohorts generally ranging from 0.726 to 0.862 [15,16,26,27,28]. While some of these approaches demonstrate better predictive efficiency, they often rely on complex feature extraction, specialized sequences, or extensive post-processing, which may limit routine clinical implementation. In comparison, the diagnostic performance of the present DECT-based model is moderate but comparable, with the advantage of simpler parameter extraction and higher workflow compatibility. Importantly, the goal of this study was not to outperform MRI or radiomics-based methods, but to explore the feasibility of using routinely acquired DECT parameters as imaging surrogates for GPC3 expression. From a clinical perspective, the proposed model may serve as a triage or risk stratification tool. For instance, in patients with unresectable HCC who are potential candidates for GPC3-targeted therapies or clinical trials, a higher predicted probability of GPC3 positivity on DECT could support prioritization for biopsy or molecular confirmation. On the other hand, in patients with contraindications to biopsy or nondiagnostic tissue samples, DECT-based prediction may provide supplementary information to guide further evaluation.

This study has several limitations. First, it was conducted at a single center with a relatively small sample size, and no formal internal validation was performed. As such, the proposed predictive model should be regarded as exploratory and hypothesis-generating, and its performance may be subject to overfitting. Internal validation and external multicenter validation are required to confirm its robustness. Second, the study population was predominantly hepatitis B virus–related HCC, which reflects regional disease characteristics. This etiological predominance may limit the generalizability of our findings to HCC populations with different underlying causes, such as hepatitis C virus infection, alcohol-related liver disease, or metabolic dysfunction–associated steatotic liver disease. Third, although efforts were made to standardize ROIs placement, manual segmentation may introduce interobserver variability, especially in necrotic or heterogeneous tumors. Future studies incorporating automated or deep learning-based segmentation could enhance measurement reliability. Finally, we dichotomized GPC3 expression, potentially oversimplifying its biological continuum. Quantitative correlation between imaging metrics and expression levels may yield more nuanced insights.

## 5. Conclusions

Quantitative parameters derived from arterial phase DECT, particularly NID and peritumoral enhancement, showed promise in predicting GPC3 expression status in HCC, with promising diagnostic performance and clinical feasibility. DECT represents a noninvasive, reproducible, and workflow-compatible imaging modality that could serve as a valuable adjunct for molecular subtyping of HCC. Future studies should leverage multicenter datasets, radiomics, and artificial intelligence to further optimize predictive modeling and advance the clinical utility of imaging biomarkers for GPC3-targeted precision therapies.

## Figures and Tables

**Table 1:** Clinicopathological characteristics of hepatocellular carcinoma patients according to GPC3 expression.

Variables	GPC3 (+) (*n* = 56)	GPC3 (−) (*n* = 23)	*p-*Value
Age (years)	59.0 (51.5, 64.0)	60.0 (53.0, 65.0)	0.795
Gender, *n* (%)			0.759
Male	48 (85.71)	21 (91.30)	
Female	8 (14.29)	2 (8.70)	
Etiology, *n* (%)			0.997
Hepatitis B	48 (85.71)	19 (82.61)	
Others	8 (14.29)	4 (17.39)	
Cirrhosis, *n* (%)			0.502
Absent	29 (51.79)	10 (43.48)	
Present	27 (48.21)	13 (56.52)	
Tumor size (cm), *n* (%)			0.861
≥5 cm	28 (50.0)	12 (52.17)	
<5 cm	28 (50.0)	11 (47.83)	
Edmondson-Steiner grade, *n* (%)			0.450
I–II	24 (42.86)	12 (52.17)	
III–IV	32 (57.14)	11 (47.83)	
Macrovascular invasion, *n* (%)			0.639
Absent	41 (73.21)	18 (78.26)	
Present	15 (26.79)	5 (21.74)	
ALT	32.0 (21.5, 60.75)	26.0 (16.0, 50.0)	0.216
AST	40.5 (29.0, 67.0)	35.0 (26.0, 52.0)	0.406
TBIL	13.85 (10.98, 18.98)	13.7 (9.10, 20.90)	0.931
ALB	36.94 ± 4.34	36.77 ± 5.87	0.885
SCr	68.48 ± 15.38	63.126 ± 10.158	0.129
PLT	153.0 (107.0, 221.0)	169.0 (106.0, 214.0)	0.901
INR	1.09 (1.03, 1.135)	1.12 (0.97, 1.19)	0.910
PT	13.5 (13.2, 14.28)	13.5 (13.00, 14.8)	0.884
AFP, *n* (%)			0.057
<10 ng/mL	21 (37.50)	14 (60.87)	
≥10 ng/mL	35 (62.50)	9 (39.13)	

GPC3, glypican-3; ALT, alanine aminotransferase; AST, aspartate aminotransferase; TBIL, total bilirubin; ALB, albumin; SCr, serum creatinine; PLT, platelet count; INR, international normalized ratio; PT, prothrombin time; AFP, alpha-fetoprotein.

**Table 2:** Qualitative CT imaging features of hepatocellular carcinoma according to GPC3 expression.

Characteristics	GPC3 (+) (*n* = 56)	GPC3 (−) (*n* = 23)	*p-*Value
Arterial phase enhancement, *n* (%)			1.000
Absent	2 (3.57)	0 (0.00)	
Present	54 (96.43)	23 (100.00)	
Internal artery, *n* (%)			0.702
Absent	27 (48.21)	10 (43.48)	
Present	29 (51.79)	13 (56.52)	
Non-peripheral washout, *n* (%)			0.121
Absent	21 (37.50)	13 (56.52)	
Present	35 (62.50)	10 (43.48)	
Enhancing capsule, *n* (%)			0.933
Absent	46 (82.14)	18 (78.26)	
Present	10 (17.86)	5 (21.74)	
Targetoid appearance, *n* (%)			1.000
Absent	48 (85.71)	20 (86.96)	
Present	8 (14.29)	3 (13.04)	
Mosaic architecture, *n* (%)			0.639
Absent	15 (26.79)	5 (21.74)	
Present	41 (73.21)	18 (78.26)	
Nodule in nodule, *n* (%)			0.997
Absent	48 (85.71)	19 (82.61)	
Present	8 (14.29)	4 (17.39)	
Blood products in mass, *n* (%)			1.000
Absent	47 (83.93)	20 (86.96)	
Present	9 (16.07)	3 (13.04)	
Margin, *n* (%)			0.775
Smooth	20 (35.71)	9 (39.13)	
Non-smooth	36 (64.29)	14 (60.87)	
Peritumoral enhancement, *n* (%)			0.029
Absent	38 (67.86)	21 (91.30)	
Present	18 (32.14)	2 (8.70)	
Necrosis or severe ischemia, *n* (%)			0.849
Absent	10 (17.86)	3 (13.04)	
Present	46 (82.14)	20 (86.96)	

GPC3, glypican-3.

**Table 3:** Quantitative parameters derived from Dual-Energy CT for GPC3 (+) versus GPC3 (-) in patients with hepatocellular carcinoma.

Parameters	GPC3 (+) (*n* = 56)	GPC3 (−) (*n* = 23)	*p-*Value
AP			
ID_Ca_	1.44 (0.94, 1.90)	0.72 (0.50, 1.45)	<0.001
ID_aorta_	8.62 (7.43, 10.05)	7.14 (6.44, 9.39)	0.062
NID (%)	17.24 (11.51, 21.74)	10.44 (7.00, 15.55)	0.001
λ_HU_	1.79 (1.18, 2.35)	0.90 (0.63, 1.80)	<0.001
Z_eff_	8.11 (7.85, 8.32)	7.72 (7.58, 8.12)	<0.001
PVP			
ID_Ca_	1.81 ± 0.63	1.48 ± 0.51	0.028
ID_aorta_	3.54 ± 0.82	3.40 ± 0.64	0.453
NID (%)	49.95 (38.66, 63.53)	44.47 (32.58, 53.26)	0.077
λ_HU_	2.24 ± 0.779	1.83 ± 0.63	0.028
Z_eff_	8.27 ± 0.29	8.11 ± 0.25	0.024

GPC3, glypican-3; AP, arterial phase; PVP, portal venous phase; ID_Ca_, iodine density of HCC; ID_aorta_, iodine density of aorta; NID, normalized iodine density; λ_HU_, slope of spectral attenuation curve; Z_eff_, effective atomic number.

**Table 4:** Univariate and multivariate logistic regression analyses for clinical characteristics, CT imaging features, and dual-energy CT parameters.

Variables	Univariable Analysis		Multivariable Analysis	
	OR (95% CI)	*p-*Value	OR (95% CI)	*p-*Value
Age	0.84 (0.60–1.18)	0.320		
Gender	2.59 (0.96–7.03)	0.061		
HBV infection	0.99 (0.95–1.04)	0.760		
Cirrhosis	0.57 (0.11–2.92)	0.502		
Tumor size	1.26 (0.34–4.69)	0.727		
Edmondson-Steiner grade	0.72 (0.27–1.90)	0.503		
Macrovascular invasion	0.92 (0.35–2.42)	0.861		
ALT	1.45 (0.55–3.85)	0.451		
AST	1.32 (0.42–4.18)	0.640		
TBIL	1.01 (0.99–1.02)	0.314		
ALB	1.00 (1.00–1.01)	0.511		
SCr	1.00 (0.96–1.04)	0.903		
PLT	1.01 (0.91–1.12)	0.883		
INR	1.03 (0.99–1.07)	0.131		
PT	1.00 (1.00–1.01)	0.822		
AFP	0.23 (0.01–4.86)	0.346		
Non-peripheral washout	2.17 (0.81–5.81)	0.124		
Enhancing capsule	0.78 (0.23–2.61)	0.690		
Targetoid appearance	1.11 (0.27–4.62)	0.885		
Mosaic architecture	0.76 (0.24–2.41)	0.640		
Nodule in nodule	0.79 (0.21–2.94)	0.727		
Blood products in mass	1.28 (0.31–5.22)	0.734		
Margin	0.86 (0.32–2.35)	0.775		
Peritumoral enhancement	4.97 (1.05–23.55)	0.043	9.25 (1.04–82.60)	0.046
Necrosis or severe ischemia	0.69 (0.17–2.78)	0.089		
Internal artery	0.83 (0.03–2.19)	0.702		
ID_Ca_-AP	5.34 (1.86–15.35)	0.002		
ID_aorta_-AP	1.23 (0.94–1.62)	0.136		
NID-AP	1.16 (1.06–1.27)	0.002	2.00 (1.18–3.40)	0.010
λ_HU_-AP	2.82 (1.36–5.87)	0.005		
Z_eff_-AP	27.34 (3.79–197.49)	0.001		
ID_Ca_-PVP	2.75 (1.08–6.96)	0.033		
ID_aorta_-PVP	1.28 (0.68–2.43)	0.448		
NID-PVP	1.02 (0.99–1.06)	0.123		
λ_HU_-PVP	2.27 (1.07–4.81)	0.033		
Z_eff_-PVP	8.62 (1.25–59.65)	0.029		

OR, odds ratio; CI, confidence interval; HBV, hepatitis B virus; AP, arterial phase; PVP, portal venous phase; ALT, alanine aminotransferase; AST, aspartate aminotransferase; TBIL, total bilirubin; ALB, albumin; SCr, serum creatinine; PLT, platelet count; INR, international normalized ratio; PT, prothrombin time; AFP, alpha-fetoprotein; ID_Ca_, iodine density of HCC; ID_aorta_, iodine density of aorta; NID, normalized iodine density; λ_HU_, slope of spectral attenuation curve; Z_eff_, effective atomic number.

**Table 5:** Diagnostic efficiency of DECT parameters in predicting GPC3 expression for HCC patients.

	AUC (95% CI)	Sen (%)	Spe (%)	*p*-Value
ID_Ca_-AP	0.774 (0.666–0.860)	89.29	60.87	<0.001
NID-AP	0.745 (0.634–0.836)	67.86	73.91	<0.001
λ_HU_-AP	0.775 (0.667–0.861)	89.29	60.87	<0.001
Z_eff_-AP	0.772 (0.664–0.859)	89.29	60.87	<0.001
ID_Ca_-PVP	0.645 (0.529–0.750)	42.86	86.96	0.027
λ_HU_-PVP	0.645 (0.529–0.749)	42.86	86.96	0.027
Z_eff_-PVP	0.647 (0.531–0.751)	42.86	86.96	0.025
PE	0.617 (0.501–0.724)	32.14	91.30	0.007
Combined model	0.781 (0.674–0.867)	67.86	78.26	<0.001

AUC, Area under the ROC curve; CI, confidence interval; Sen, sensitivity; Spe, specificity; AP, arterial phase; PVP, portal venous phase; ID_Ca_, iodine density of HCC; NID, normalized iodine density; λ_HU_, slope of spectral attenuation curve; Z_eff_, effective atomic number; PE, peritumoral enhancement; Combined model, the model combining NID-AP and peritumoral enhancement.

## Data Availability

The original contributions presented in this study are included in the article/Supplementary Material. Further inquiries can be directed to the corresponding author.

## Supplementary Materials

The following supporting information can be downloaded at: https://www.mdpi.com/article/10.3390/diagnostics16060850/s1, Table S1: Definition of CT imaging features of HCC according to LI-RADS 2018; Table S2: ICCs of the measurement data between the two radiologists.

## Abbreviations

The following abbreviations are used in this manuscript:

AP	arterial phase
AUC	area under the curve
DECT	dual-energy computed tomography
GPC3	glypican-3
ID_Ca_	iodine density of HCC
ICC	intraclass correlation coefficients
MRI	magnetic resonance imaging
NID	normalized iodine density
OR	odds ratio
PET	positron emission tomography
PVP	portal venous phase
ROC	receiver operating characteristic
Z_eff_	effective atomic number
λ_HU_	slope of spectral attenuation curve
