# Supplementary material for: Dual-Energy CT-Derived Parameters: A Promising Tool for Noninvasive Prediction of Glypican-3 in Hepatocellular Carcinoma
Source: Diagnostics (Basel). 2026 Mar 12;16(6):850. doi: 10.3390/diagnostics16060850 (PMC13025459; doi:10.3390/diagnostics16060850)
Supplement: Supplementary file 1 [file diagnostics-16-00850-s001.zip › diagnostics-4131620-supplementary.pdf]

Table S1. Definition of CT imaging features of HCC according to LI-RADS 2018.

CT imaging features of HCC	Definition
Arterial Phase Hyperenhancement (APHE)	The lesion shows hyperenhancement in the arterial phase relative to the surrounding liver parenchyma, excluding peripheral rim enhancement. This reflects neovascularity typical of HCC.
Nonperipheral Washout	The lesion becomes hypointense or hypodense relative to the liver in the portal venous or delayed phases, with a non-peripheral distribution, indicating specific hemodynamics seen in HCC.
Enhancing Capsule	A smooth, uniform, enhancing rim seen in the portal venous, delayed, or transitional phase, representing either a fibrous capsule or pseudocapsule. It is a hallmark of progressed HCC.
Targetoid Appearance	Lesion shows rim arterial phase hyperenhancement and delayed central enhancement, creating a "target" appearance. This is commonly associated with non-HCC malignancies such as cholangiocarcinoma.
Mosaic Architecture	The lesion contains multiple compartments with different signal intensities or densities, giving it a "mosaic" pattern. It reflects heterogeneity and multiclonality, often in large HCCs.
Nodule-in-Nodule Pattern	One or more smaller nodules are seen within a larger lesion, with different imaging characteristics, suggesting dedifferentiation within the tumor. Common in HCC development.
Intratumoral Hemorrhage	Areas of high signal or density within the tumor indicate internal bleeding. This suggests aggressive behavior and is frequently observed in HCC.
Tumor Margin	The edge morphology of the lesion may offer diagnostic clues—HCC often has smooth or lobulated margins, whereas non-HCC malignancies may have irregular or infiltrative margins.
Peritumoral Enhancement	Enhancement surrounding the tumor during arterial or portal venous phase, also known as "corona enhancement." It may indicate aggressive vascular behavior.
Necrosis or Severe Ischemia	Non-enhancing areas within the tumor suggest necrosis or significant ischemia, commonly seen in non-HCC malignancies like intrahepatic cholangiocarcinoma.
Internal Artery	Arterial vessels seen within the lesion, reflecting rich blood supply. This supports a diagnosis of HCC.

Table S2. ICCs of the measurement data between the two radiologists.

Parameters	ICC	95% CI	*p*-value
ID_{Ca}-AP	0.934	0.879-0.964	< 0.001
ID_{aorta}-AP	0.886	0.796-0.938	< 0.001
NID-AP	0.808	0.668-0.893	< 0.001
λ_{HU}-AP	0.934	0.878-0.964	< 0.001
Z_{eff}-AP	0.928	0.869-0.961	< 0.001
ID_{Ca}-PVP	0.788	0.633-0.882	< 0.001
ID_{aorta}-PVP	0.971	0.943-0.985	< 0.001
NID-PVP	0.776	0.615-0.875	< 0.001
λ_{HU}-PVP	0.787	0.632-0.882	< 0.001
Z_{eff}-PVP	0.787	0.631-0.881	< 0.001

ICC, intraclass correlation coefficient; CI, confidence interval; AP, arterial phase; PVP, portal venous phase; ID_{Ca}, iodine density of HCC; ID_{aorta}, iodine density of aorta; NID, normalized iodine density; λ_{HU}, slope of spectral attenuation curve; Z_{eff}, effective atomic number.
